# ﻿Speciation and diversification of the *Bupleurum* (Apiaceae) in East Asia

**DOI:** 10.3897/phytokeys.248.132707

**Published:** 2024-10-22

**Authors:** Yong-xiu Song, Ceng-yue Yang, Yu-Yang Zhou, Yan Yu

**Affiliations:** 1 Key Laboratory of Bio-Resources and Eco-Environment of Ministry of Education, College of Life Sciences, Sichuan University, Chengdu 610064, China Sichuan University Chengdu China

**Keywords:** *Bupleurum*, museum model, phylogeny, species diversity

## Abstract

*Bupleurum*, belonging to the Apiaceae, is widely distributed across the Eurasian continent. The origin and species diversification of *Bupleurum* in East Asia, remain incompletely resolved due to the limited samples in previous studies. To address these issues, we have reconstructed a robust phylogenetic framework for *Bupleurum* in East Asia based on the ITS and three plastid genes. Our phylogenetic analysis confirms the monophyly of *Bupleurum* with strong support. Both ITS and chloroplast dataset divided the *Bupleurum* in East Asia into East Asia Group I and East Asia Group II in this study. The divergence time and ancestral area reconstruction of ITS dataset indicated that the *Bupleurum* originated in the Mediterranean basin and its adjacent areas around 50.33 Ma. subg. Penninervia and subg. Bupleurum diverged at about 44.35 Ma, which may be related to the collision of India with the Eurasian continent. Both East Asia Group I and East Asia Group II originated from a common ancestor in the Mediterranean, East Asia Group I divergence around 12.95 Ma; East Asia Group II divergence around 13.32 Ma. The character reconstruction showed that the morphological characters and altitude distribution analyzed in this study exhibit a scattered distribution in East Asian Group I and East Asian Group II. Additionally, diversification rate analysis shows that the East Asian Group I and East Asian Group II exhibited no significant shifts in diversification rates in the evolutionary history according to ITS and combined dataset. Both molecular and morphological data supports that East Asian *Bupleurum* is a museum taxon, meaning that the species diversity of East Asian *Bupleurum* has gradually accumulated over time.

## ﻿Introduction

Understanding the spatiotemporal distribution patterns of species, along with their diversification mechanisms (i.e., the historical process by which the same ancestor evolved successively through time and space to produce existing species) has long been an important issue in evolutionary biology and ecology (Shrestha et al. 2018). *Bupleurum* L. stands out as a uniquely distinct group within the Apiaceae characterized by its simple, entire leaves. It comprises about 180–200 species, of which about 50 are found in East Asia (Linczevski 1950; Sheh and Watson 2005). This genus is widely distributed in the North temperate regions of Eurasia, with two exceptions: *B.americanum* J. M. Coult. and Rose in North America and *B.mundii* Cham. and Schltdl. in South Africa (Neves and Watson 2004). *Bupleurum* has two recognized hotspots of diversity: the Mediterranean Basin and the Himalaya-Hengduan Mountains region (Neves and Watson 2004; Huang et al. 2021a). *Bupleurum* possesses a rich morphological and ecological diversity, ranging from annual to perennial herbs, and even includes subshrubs, shrubs, such as *B.gibraltaricum* and *B.fruticosum* native to the Mediterranean and adjacent areas, and *B.dracaenoides* endemic to the Hengduan Mountains (Wang et al. 2011; Huang et al. 2021b).

Molecular phylogenetics evidence supports *Bupleurum* as a monophyletic group (Neves and Watson 2004; Wang et al. 2008, 2011; Banasiak et al. 2013; Wen et al. 2021). Combining morphological and phylogenetic data, existing studies have divided the *Bupleurum* genus into two subgenera: subg. Penninervia and subg. Bupleurum (Neves and Watson 2004). Subg. Penninervia includes the Mediterranean woody and perennial species characterized by pinnate reticulate veins; while subg. Bupleurum comprises the majority of species, distinguished by their parallel veins. Furthermore, Neves and Watson (2004) based on ITS or a few plastid markers have hypothesized that the *Bupleurum* originated in the western Mediterranean. *Bupleurum* in East Asia have been divided into two groups: East Asia Group I and East Asia Group II (Wang et al. 2008, 2011; Ma 2011; Ma et al. 2013, 2014). The two East Asia groups of the *Bupleurum* are believed to have originated from species near the Mediterranean, subsequently migrating eastward through the Middle East and the Caucasus to East Asia (Neves and Watson 2004; Wang et al. 2008, 2011). Moreover, a genealogical-geographical study of *B.longiradiatum* by Zhao et al. (2013). showed that *B.longiradiatum* is a genetically diverse species, with two corresponding refugia found throughout its distribution range.

Thus, as a monophyletic group broadly distributed across the Eurasian continent, the *Bupleurum*, with its rich species diversity, serves as an excellent subject for studying patterns in species richness distribution. Previous research on East Asian *Bupleurum* has primarily focused on taxonomy, phylogenetics, and phylogeography (Wang et al. 2011; Huang et al. 2021b). There is a lack of research on the spatiotemporal distribution patterns and species diversification within East Asian *Bupleurum*.

Here, we collected ITS and three plastid genes (matK, psbA - trnH, and rbcL) for 89 species of *Bupleurum* as a source of phylogenetic information; The purpose of our study was to (1) reconstruct a phylogenetic framework of the East Asian *Bupleurum*, and reconstruct the ancestral distribution of the East Asian *Bupleurum* to explore its origins and dispersal processes; and (2) perform divergence time estimation and diversification rate analysis, revealing the species formation patterns of East Asian *Bupleurum*.

## ﻿Materials and methods

### ﻿Materials collection and sequencing

89 *Bupleurum* representing all major branches within the *Bupleurum* were used for the phylogenetic analysis in this study. Of these, 25 species were newly collected from the wild. The fresh leaves were collected and preserved in silica gel. Voucher specimens were collected and deposited in Sichuan University Herbarium (SZ). (Suppl. material 1: table S1). 61 ITS sequences and three plastid genes (56 matK, 49 psbA - trnH, and 49 rbcL) of *Bupleurum* were downloaded from NCBI. The species names and GenBank accession numbers are listed in Suppl. material 1: table S2. The downloaded sequences were used as a reference to extract the ITS sequences and chloroplast genes of the corresponding species from the next-generation sequencing data of 25 newly sequenced species and the raw sequencing da-ta of 9 *Bupleurum* species (5 from the SSR library and 4 from the study by Huang et al. (2021a) using GeneMiner (Xie et al. 2024). ITS and three chloroplast genes from 34 *Bupleurum* species were extracted using GeneMiner. A total of 86 ITS, 66 matK, 64 psbA-trnH, and 64 rbcL were used for phylogenetic analysis.

### ﻿Phylogeny reconstruction

Species from the Apioideae: *Chamaesiumnovemjugum*, *C.mallaeanum*, *C.wolffianum*, *C.thalictrifolium*, *C.spatuliferum*, *C.delavayi* and species from the Saniculoideae: *Saniculaastrantiifolia*, *S.canadensis* were used as outgroup. ITS dataset and plastid genes dataset were aligned using MAFFT (Katoh and Standley 2013) and then trimmed using TrimAl v1.2 (Capella-Gutierrez et al. 2009). Due to the limited number of parsimony-informative sites in plastid genes, which increases the potential for gene tree errors, we inferred the plastid species trees using the concatenation method, applying both Maximum Likelihood (ML) and Bayesian Inference (BI) techniques. ITS, matK, psbA-trnH, and rbcL sequences were concatenated using PhyloSuite (Zhang et al. 2020). ModelFinder (Kalyaanamoorthy et al. 2017) was used to construct and determine the best-fitting nucleotide substitution models for each dataset. The maximum likelihood (ML) analyses of the above datasets were using FastTree 2.1 (Price et al. 2010) performing 10000 bootstrap replicates with the GTR + G model. The MrBayes v3.2.7 (Ronquist et al. 2012) was selected for Bayesian analysis under GTR + I + G model. Two independent Markov chain Monte Carlo (MCMC) runs were performed, each with two chains of 10000000 generations, of which every 1000 generation was sampled. After discarding the first 25% trees as the burn-in, a consensus tree of the remaining trees was produced.

### ﻿Estimation of divergence time

Divergence time of *Bupleurum* was estimated with a lognormal relaxed molecular clock model in BEAST v1.10.4 (Drummond and Rambaut 2007). The pollen fossil adopted by (Banasiak et al. 2013) was used to determine species node priors. The calibration point was placed at the stem node of Bupleureae (Gruascavagnetto and Cerceaularrival 1984) with a lower bound of 33.90 Mya (the end of the Priabonian) and the upper bound of 58.7 Mya (the beginning of the Thanetian). The BEAST analysis was run for 100 million generations sampling every 10000 generations. The GTR + G substitution model was selected and a Yule tree prior was used for the analysis. The stationarity of the chains and convergence of two runs was monitored by Trace v1.7 (Rambaut et al. 2018), with the effective sample size of all parameters > 200.

### ﻿Reconstruction of ancestral area

The following seven regions were defined for biogeographic analyses based on the natural geography and climatic history and also according to the distribution of *Bupleurum*: (A) the Mediterranean Basin, North Africa, and Europe; (B) Central Asia and West Asia; (C) the Eastern Himalayas - Hengduan Mountains region and South Asia; (D) East Asia; (E) North Asia; (F) North America; (G) Southern Africa. The distribution areas of the study species were determined based on GBIF (2023), POWO (2024) and the WFO (2024), and field observations. Reconstructions of the ancestral area of *Bupleurum* were conducted using the Bayesian Binary MCMC as implemented in the RASP 4 (Yu et al. 2020). We used the divergence times tree based on ITS for Bayesian Binary MCMC analysis. We removed outgroups before ancestral-state reconstruction to avoid biased inferences for the crown node of the ingroups, which could arise from uncertainty in the root areas of an outgroup.

### ﻿Morphological character evolution

We collected 7 characters (key taxonomic traits for identifying species of the *Bupleurum* in the WFO (2024)) and altitude distribution for 55 species of the *Bupleurum*. The above ITS divergence time tree was used for character evolution analysis after removing the outgroups and the species with extensive missing morphological data. We conducted the reconstruction of an ancestral trait of East Asian *Bupleurum* using MultiState Reconstruction with the Bayes Traits method implemented in RASP 4 (Yu et al. 2020). All the characters were treated as unordered and equally weighted. These morphological characters and altitudes were mapped and coded in Table 1. The matrix for the East Asian *Bupleurum* was compiled based on specimens, available literature, and databases (such as GBIF 2023; WFO 2024), and is presented in Suppl. material 1: table S3.

**Table 1.** Coding of morphological characters.

Character	Character states
Plant height	A < 50 cm; B ≥ 50 cm
Stem base with fibrous remnant sheaths	A no; B yes
Rays	A < 3; B ≥ 3 < 6; C ≥ 6
Number of bracteoles	A < 5; B ≥ 5
Shape of bracteoles	A ovate (broadly ovate, obovate); B elliptic; C lanceolate (narrowly lanceolate, ovate-lanceolate); D linear; E suborbicular
Petals color	A yellow; B purple; C green;
Vittae in each furrow	A < 3; B ≥ 3
Altitudes	A < 1000 m; B 1000–3000 m; C > 3000 m

### ﻿Diversification rate analysis

Time-calibrated tree based on the ITS and the combined ITS and chloroplast dataset of the *Bupleurum* were used for diversification rate analysis in BAMM (Shi and Rabosky 2015). After removing the outgroups, we ran 10000000 generations of MCMC, discarding the first 10% as burn-in and conducted analysis and plotting using the BAMMtools (Rabosky et al. 2014). Additionally, we used MEDUSA (Alfaro et al. 2009) to estimate shifts in diversification rates. After removing the outgroups, the tree was imported into the R package MEDUSA for computation.

## ﻿Results

### ﻿Gene extraction and phylogenetic analyses

**Figure 1.**
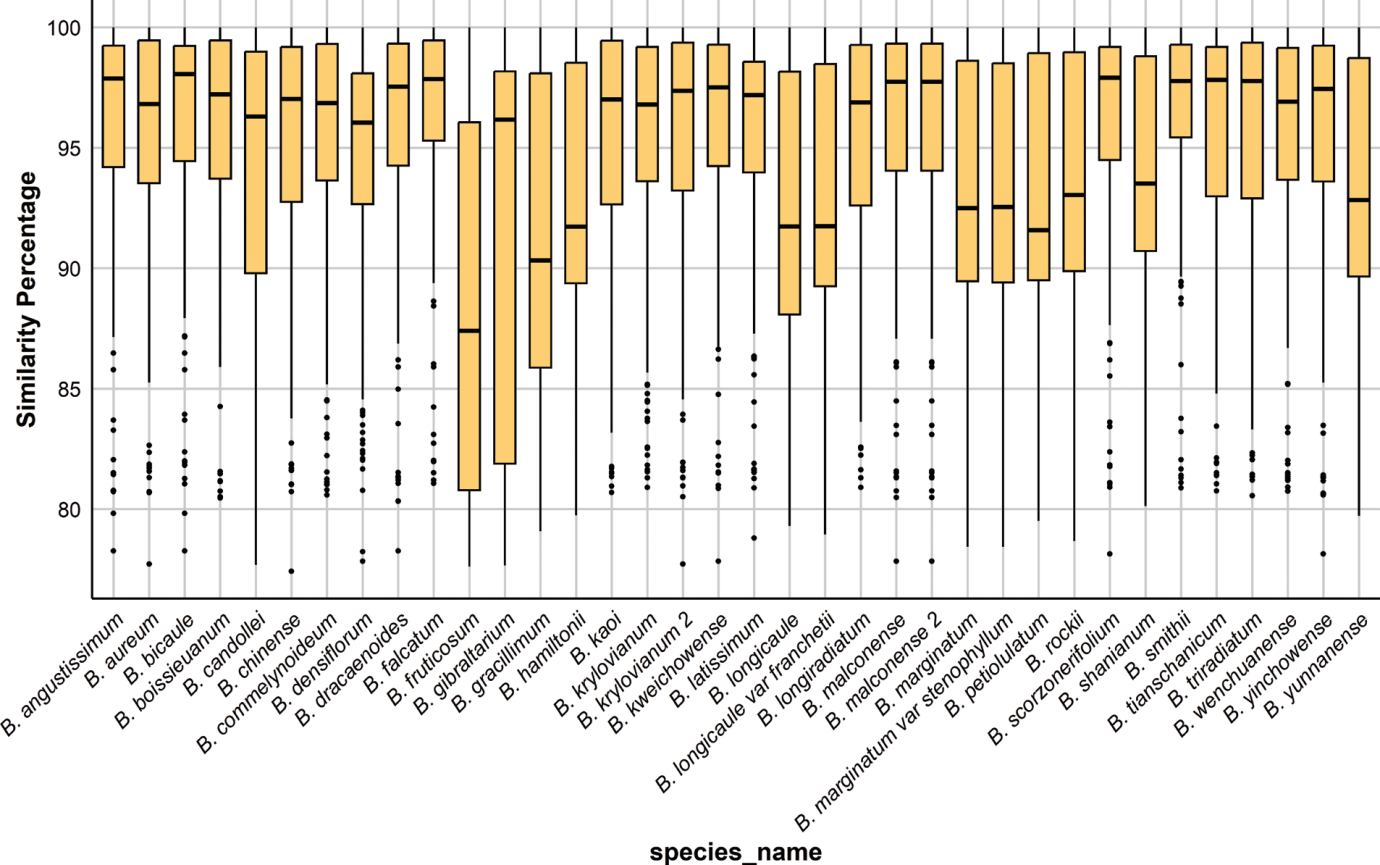
Box plot of the results of assembling ITS and three chloroplast genes using GeneMiner, with the x-axis representing sample names and the y-axis representing sequence similarity (%).

The downloaded ITS and chloroplast sequence data were used as reference se-quences to evaluate the assembly results based on sequence similarity. The median se-quence similarity for all samples, except *Bupleurumfruticosum*, was above 90%, indicating a high level of reliability (Fig. 1, Suppl. material 1: table S4).

**Figure 2.**
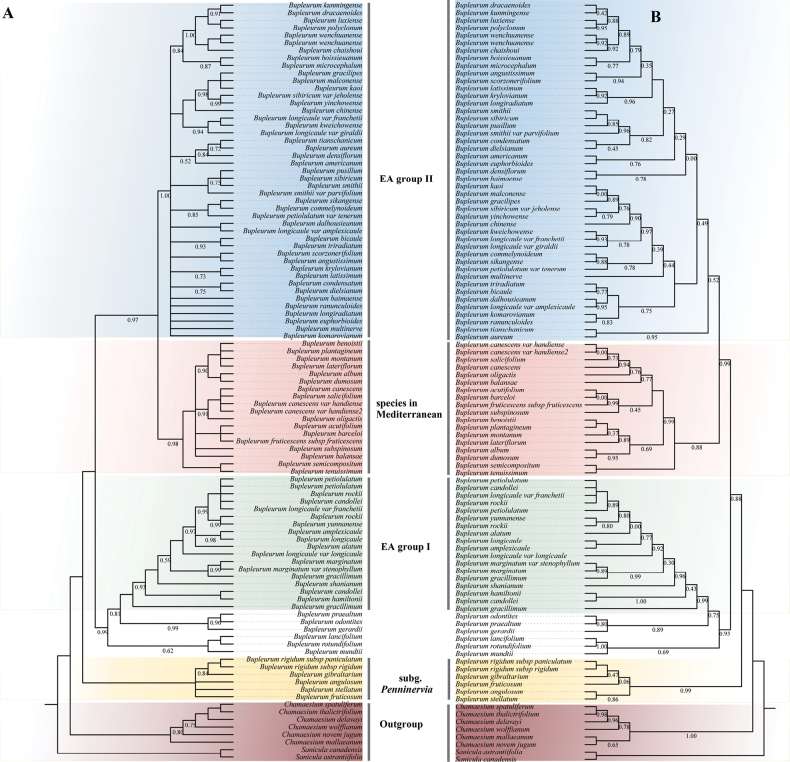
Phylogenetic relationships of *Bupleurum* inferred from ITS based on BI methods and ML methods. PP (posterior probability) values and BS (bootstrap) values are shown above the branches (only PP < 1.0 are shown) **A** tree topology inferred by BI methods **B** tree topology inferred by ML methods.

The ITS and the three plastid genes recognized the *Bupleurum* as monophyletic with robust support (PP = 1.00, BS = 100%) (Fig. 2, Suppl. material 2: fig. S1). Two major groups were recovered within East Asian *Bupleurum*, EA Group I and EA Group II. According to ITS dataset, EA Group I is sister to *Bupleurumodontites*, *Bupleurumpraealtum*, *Bupleurumgerardii*; EA Group II is sister to clade of species in Mediterranean.

### ﻿Estimation of divergence times

**Figure 3.**
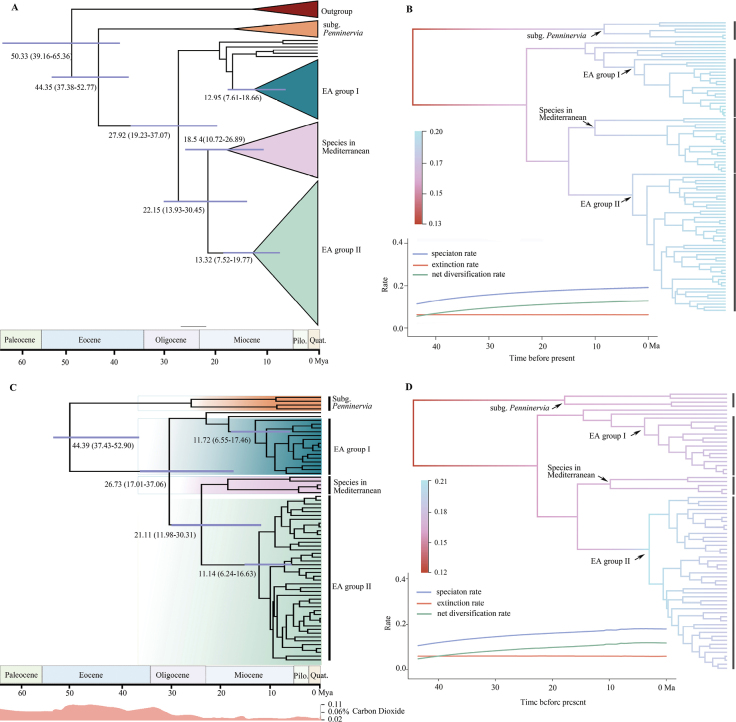
The estimation of divergence time and diversification rate analysis of *Bupleurum***A, B** ITS **C, D** ITS + matK + psbA - trnH + rbcL **A, C** estimation of divergence time of Bupleurum, the 95% highest posterior density (HPD) estimates for each well-supported clade are represented by bars, historical carbon dioxide levels data from TimeTree **B, D** diversification rate analysis of *Bupleurum*.

Divergence time analyses based on the combined dataset are basically consistent with the ITS dataset of the *Bupleurum* (Fig. 3a, c). According to the ITS dataset, the stem age of *Bupleurum* was estimated as ca. 50.33 Ma (95% HPD: 39.16–65.36 Ma). The crown group of this genus was dated to the middle Eocene (ca. 44.35 Ma; 95% HPD: 37.38–52.77 Ma) and then diverged into two major clades (subgen. Penninervia and subgen. Bupleurum, Fig. 3a). The crown of the subgen. Bupleurum was dated to the middle Oligocene (ca. 27.92 Ma; 95% HPD: 19.23–37.07 Ma); and the crown of EA Group I is predicted to have originated at ca.12.95 Ma (95% HPD: 7.61–18.66 Ma), and EA Group II originated at ~13.32 Ma (95% HPD: 7.52–19.77 Ma).

### ﻿Reconstruction of ancestral area

**Figure 4.**
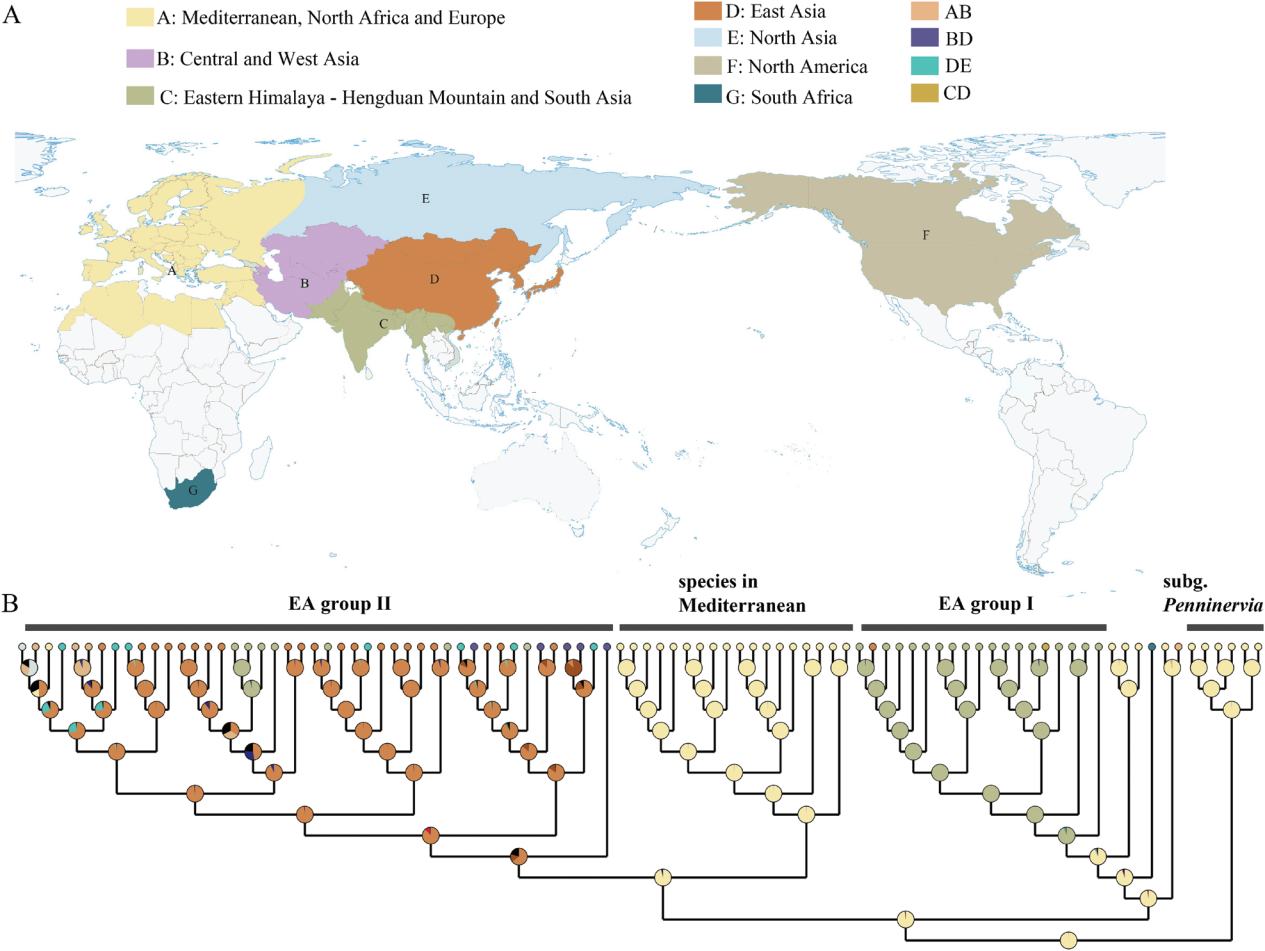
Reconstructions of the ancestral area based on ITS-based phylogeny of *Bupleurum*. node pie show the inferred ancestral ranges, pie colors to regions defined in the caption and world map.

The reconstructions of the ancestral area based on the ITS dataset (Fig. 4) supported the most likely ancestral distribution of *Bupleurum* as being in the Mediterranean Basin, which served as a site of diversification. Subgen. Penninervia and subgen. Bupleurum diverged in the mid-Eocene, approximately 44.35 Ma. Subg. Bupleurum experienced its first divergence at around 27.92 Ma, where one of its clades, diverging at 12.95 Ma, spread to the Qinghai-Tibet Plateau - Hengduan Mountains region, forming the East Asia Group I. The other branch diverged in the Mediterranean basin around 22.15 Ma, with one clade remaining in the Mediterranean. Meanwhile, another clade dispersal occurred from Central Asia to East Asia, forming the East Asia Group II.

### ﻿Character evolution

**Figure 5.**
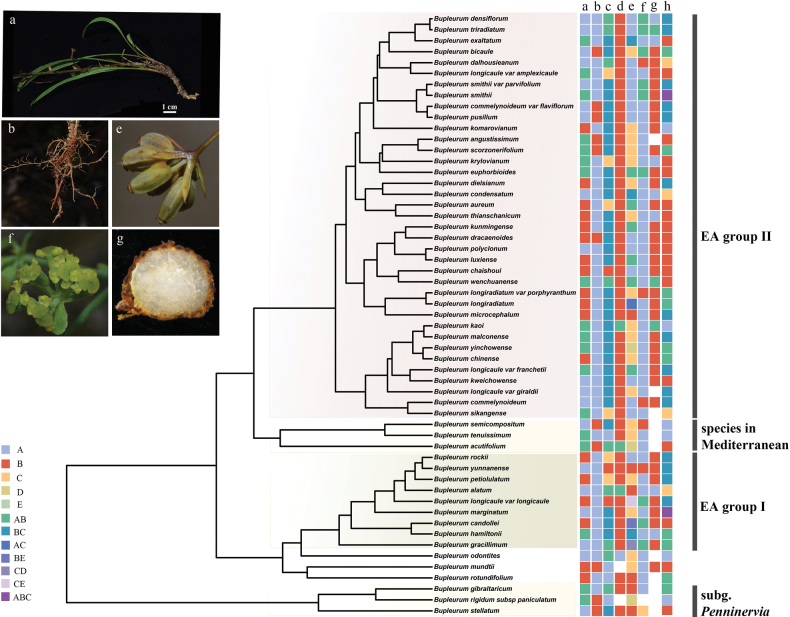
Reconstruction of the ancestral trait of the morphological character for *Bupleurum***a–h** represent different traits and different colors represent different trait states **a** plant height **b** stem base with fibrous remnant sheaths **c** rays **d** number of bracteoles **e** shape of bracteoles **f** petals color **g** vittae in each furrow **h** altitudes.

The 8 characters including macroscopic characters and altitude distribution were mapped on the phylogenetic tree to reconstruct ancestral states and analyze evolution trends. The traits examined in this study exhibit a scattered distribution across the phylogenetic tree (Fig. 5), with every observed state of the traits occurring within both the East Asian Group I and East Asian Group II. And none of the traits’ state were unique to a particular evolutionary clade. Morphological characters and altitude distribution are highly variable. The shape of the bracteoles varies widely from ovate, elliptic, lanceolate, linear, to suborbicular, and the rays varies from 2 to 18. In addition, *Bupleurum* has a wide altitudinal distribution, ranging from 100 to 4300 meters above sea level, as illustrated in Suppl. material 2: figs S2, S3, S4, S5.

### ﻿Diversification rate analysis

Diversification rate analysis of the ITS dataset and the combined dataset using BAMM yielded similar results (Fig. 3b, d). Utilizing the ITS dataset, *Bupleurum* evolved at a relatively constant rate (average speciation rate λ = 0.1888 Myr^-1^) in the evolutionary history (Fig. 3b). The net diversification rate of all *Bupleurum* species estimated by BAMM increased slowly over time with the speciation rate ranging from a minimum of λ = 0.1048 to a maximum of λ = 0.3587 Myr^-1^. The speciation rate for East Asian group I was 0.1926 Myr^-1^, and that for East Asian group II was 0.1930 Myr^-1^. The results from the MEDUSA analysis indicate that no significant shifts in diversification rate were detected in either the ITS dataset or the combined dataset.

## ﻿Discussion

### ﻿The origin and spread of the *Bupleurum* in East Asia

As a widely distributed genus within the Apiaceae, phylogenetic relationships among the main branches of *Bupleurum* have consistently attracted significant interest in previous studies (Neves and Watson 2004; Sheh and Watson 2005; Wang et al. 2008, 2011; Huang et al. 2021a). Our phylogenetic analyses of both the ITS genes’ dataset and the plastid genes dataset presented here robustly support earlier studies that *Bupleurum* is monophyletic (Wang et al. 2008, 2011). *Bupleurum* in East Asia was partitioned into two groups, namely, East Asia Group I and East Asia Group II. Species in East Asian Group I are exclusively found in the Tibetan Plateau and the Hengduan Mountains, while the species of East Asian Group II are widely distributed in the central and eastern of Asia and Europe, as well as in the northern of North America (*B.americanum*, endemic to North America).

This study conducted extensive sampling in the two distribution centers of the *Bupleurum*: the Mediterranean Basin and the Qinghai-Tibet Plateau-Hengduan Mountains region. Based on the ITS dataset, divergence time and ancestral area reconstructions for the *Bupleurum* suggest that it diverged in the Eocene (50.33 Ma) in the Mediterranean Basin (a center of diversification for many seed plants) and nearby areas, which is consistent with previous studies (Neves and Watson 2004; Banasiak et al. 2013; Wen et al. 2021; Calvino et al. 2016). In this study, the subg. Penninervia and subg. Bupleurum began to diverge at 44.35 Ma. Then, subg. Penninervia remained in the Mediterranean and nearby areas, while subg. Bupleurum spread to East Asia, forming the East Asian group. During this period, carbon dioxide levels were high, leading to a relatively warm global climate. The Indian plate and the Eurasian plate were in the midst of their collision (approximately 50–25 Ma), resulting in the rapid uplift of the Himalayas and the Tibetan Plateau. These geological events profoundly impacted the evolution of biota on the Eurasian continent. The collision between India and Eurasia, combined with the high-carbon environment, likely created new ecological niches that promoted species differentiation. This may have contributed to the divergence of the *Bupleurum*. (Aitchison et al. 2007; van Hinsbergen et al. 2012; Yang et al. 2021). The first diversification within subg. Bupleurum occurred at 27.92 Ma. One clade, diversifying at 19.8 Ma, spread to the Qinghai-Tibet Plateau - Hengduan Mountains region, forming the East Asian group I. Another clade diverged at 22.15 Ma in the Mediterranean Basin, with one subclade remaining there and another spreading from Central Asia to East Asia, forming the East Asian group II. During this period of divergence in the Oligocene, the global climate gradually cooled, and carbon dioxide levels decreased. These climatic changes likely exerted evolutionary pressure on the species, possibly leading to the divergence of subgenera of *Bupleurum*.

Fluctuations in carbon dioxide levels and significant climate changes imposed various pressures on species evolution during the Miocene epoch (23–10 Ma). Both the Himalayas and the Tianshan Mountains experienced a significant uplift, and a drying event in Central Asia led to drastic reductions in rainfall, greatly impacting many taxons (Yu et al. 2014; Yang et al. 2017; Zheng et al. 2021). Thus, the diversification of the two East Asian groups of *Bupleurum* might be related to these events. The crown of East Asian groups I and East Asian groups II are estimated to be 12.95 Ma and 13.32 Ma, respectively. After the late Miocene (10 Ma - present), the Tibetan Plateau underwent further uplift and expansion, while the Hengduan Mountains in its southeastern margin experienced intense orogenic activity. Warm, moist air from the Indian Ocean, blocked by the Himalayas and Kailash Range, entered the East Asian region through the Hengduan Mountains, bringing significant rainfall. These continuous geological uplift events further intensified the monsoon climate in East Asia, significantly impacting the regional and global climate patterns. These events collectively shaped the complex geological and geomorphological features of the Hengduan Mountains, providing diverse habitats for plants and making it one of the global biodiversity hotspots (López-Pujol et al. 2011; He and Jiang 2014; Sklenar et al. 2014; Yang et al. 2017; Xie et al. 2019). The diversification within East Asian groups I and II may have been influenced by these geographical processes. It is speculated that the species diversification of East Asian group I was mainly influenced by the dramatic uplift of the Hengduan Mountains, while the diversification of East Asian group II, originating from Central Asia, was more influenced by the monsoon climate. Field observations and herbarium records indicate that species from East Asian group II prefer drier, sunnier environments (Wang et al. 2008, 2011; Ma 2011; Ma et al. 2013, 2014), which may be related to the arid conditions of Central and West Asia. Additionally, ancestral area reconstructions based on ITS also suggest that the common ancestor of East Asian group II likely originated from Central Asia.

Interestingly, *B.americanum* (endemic to North America) is placed in East Asian group II. Phylogenetic indicate that it is closely related to *B.euphorbioides*, which is located at the eastern edge of the Eurasian continent. We speculate that the ancestor of *B.americanum* spread from the eastern edge of Eurasia to North America via the Bering Land Bridge. Meanwhile, *B.mundii*, an endemic species in South Africa, is placed in basal clades of East Asian group I. This suggests that the ancestor of *B.mundii* likely originated from the Mediterranean.

### ﻿The *Bupleurum* in East Asia is a museum taxon

Two classical models have been used to explain patterns of species diversity from an evolutionary perspective. The first model is the “evolutionary cradle” model, which emphasizes certain events in history that created ecological opportunities for ancestral species to undergo adaptive radiation. Its main characteristic is the temporal and spatial variation in diversification rates, accumulating diversity rapidly through high species formation rates (Fischer 1960). For instance, Madagascar is often considered an “evolutionary cradle” because it hosts many unique and recently evolved species (Pastorini et al. 2009; Florio et al. 2012). The second model is the “museum” model. It highlights the comparatively stable diversification rates and lower extinction rates of species over time and space, allowing species diversity to accumulate gradually. This model posits that older evolutionary branches have more species because they have had more time to accumulate diversity, and this is unrelated to changes in diversification rates (Haffer 1969; Gentry 1982; Schley et al. 2018; Loiseau et al. 2020; Vargas and Dick 2020). An example includes the Troidini butterflies of the Neotropics (Condamine et al. 2012). Overall, the former focuses more on the recent origin and rapid evolution of species, while the latter values the long-term persistence and stability of species. Ultimately, the patterns of species diversity are generated by the processes of speciation, extinction and dispersal that occur over evolutionary time-scales (Wiens and Donoghue 2004).

In our diversification rate analysis based on ITS dataset and the combined dataset for the *Bupleurum*, we evaluated whether there were differences in evolutionary rates among different clades of the East Asian *Bupleurum*. Neither BAMM nor MEDUSA analyses based on ITS detected any diversification rate shifts within the East Asian group. Given that both distribution centers of *Bupleurum* are extensively sampled and species richness is lower in other regions, it is less likely to detect changes in species diversification rates, suggesting a low probability of significant diversification rate shifts within *Bupleurum*. The character reconstruction showed that every state of traits and altitude distribution were observed within both the East Asian Group I and East Asian Group II, showing a high degree of variability and that none of the trait states were unique to a particular evolutionary clade.

Combining the results of molecular data and character evolution, it can be hypothesized that the East Asian *Bupleurum* support the “museum” model, i.e., their rate of diversification has not undergone any abrupt shifts during their long evolutionary history. More extensive research on the diversification rates of the Apioideae supports this conclusion (Baczyński et al. 2022). Although the specific estimates of diversification rates vary, unlike rapid diversification rate shifts within groups like the Scandicineae and Tordyliinae Drude, there is no shift in diversification rates within the East Asian *Bupleurum*.

## ﻿Conclusions

This paper reconstructs the phylogenetic relationships of the *Bupleurum* in East Asia based on ITS dataset and the combined dataset. The results suggest that the *Bupleurum* is a monophyletic group, and that East Asian *Bupleurum* is further divided into East Asian Group I and East Asian Group II. The study on the divergence time and ancestral area reconstruction of the *Bupleurum* indicates that it differentiated in the Mediterranean basin and nearby areas around 50.33 Ma, with two subgenera (Penninervia and subg. Bupleurum) diverging around 44.35 Ma, possibly related to the collision of India with the Eurasian continent. The speciation of East Asian Group I species might have been influenced by the dramatic uplift of the Hengduan Mountains, while the diversification of East Asian Group II could be more affected by the monsoon climate, possibly related to the arid environment of Central Asia. Additionally, the results of the diversification rate analyses based on ITS and the combined dataset, conducted using both BAMM and MEDUSA methods consistently indicated that there were no significant changes in diversification rates in the evolutionary history of the East Asian *Bupleurum*. The character reconstruction showed that every state of traits and altitude distribution were observed within both the East Asian Group I and East Asian Group II. Both molecular and morphological evidence support the East Asian *Bupleurum* as a ‘museum’ taxon.

## ﻿Additional information

### Data availability

All of the data that support the findings of this study are available in the main text or Supplementary Information.

## Supplementary materials

Supplementary material 1 Supplementary tables Data type xlsx Explanation note **table S1.** Voucher specimens in this study. **table S2**. GenBank accession numbers for *Bupleurum*. **table S3.** Character matrix in this study. **table S4.** median similarity percentage in this study. https://binary.pensoft.net/file/1157847This dataset is made available under the Open Database License (http://opendatacommons.org/licenses/odbl/1.0/). The Open Database License (ODbL) is a license agreement intended to allow users to freely share, modify, and use this Dataset while maintaining this same freedom for others, provided that the original source and author(s) are credited.Yong-xiu Song, Ceng-yue Yang, Yu-Yang Zhou, Yan Yu

Supplementary material 2 Supplementary figures Data type zip Explanation note **figure S1.** Phylogenetic relationships of Bupleurum inferred from matK + psbA – trnH + rbcL based on BI methods and ML methods. **figure S2**. Reconstruction of the ancestral trait of the morphological character for *Bupleurum*. **figure S3**. Reconstruction of the ancestral trait of the morphological character for *Bupleurum*. **figure S4**. Reconstruction of the ancestral trait of the morphological character for *Bupleurum*. **figure S5**. Reconstruction of the ancestral trait of the morphological character for *Bupleurum*. https://binary.pensoft.net/file/1157848This dataset is made available under the Open Database License (http://opendatacommons.org/licenses/odbl/1.0/). The Open Database License (ODbL) is a license agreement intended to allow users to freely share, modify, and use this Dataset while maintaining this same freedom for others, provided that the original source and author(s) are credited.Yong-xiu Song, Ceng-yue Yang, Yu-Yang Zhou, Yan Yu
