# Supplementary figures and images for: ﻿Speciation and diversification of the Bupleurum (Apiaceae) in East Asia
Source: PhytoKeys. 2024 Oct 22;248:41–57. doi: 10.3897/phytokeys.248.132707 (PMC11522740; doi:10.3897/phytokeys.248.132707)

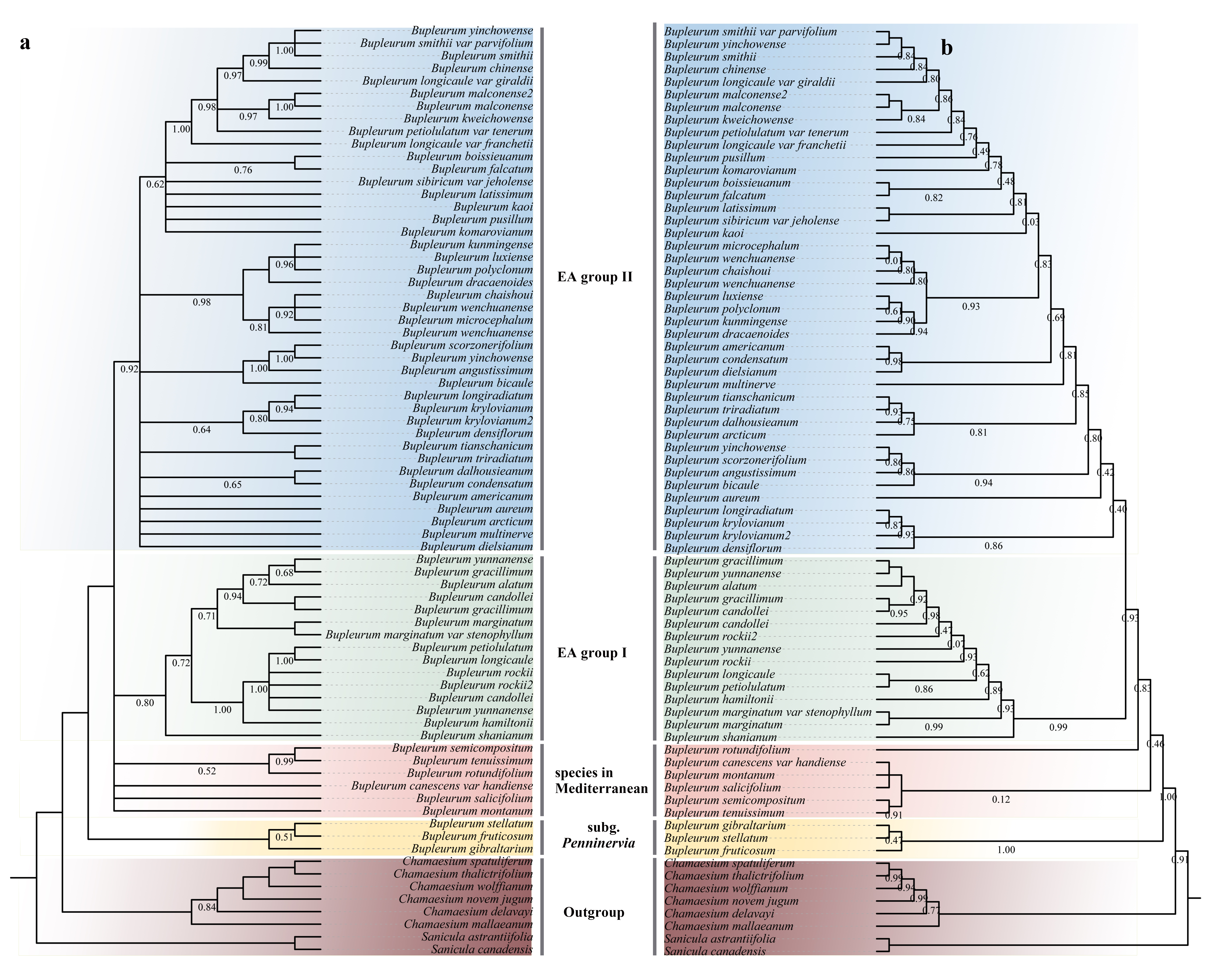

Supplement: Supplementary material 2 — Supplementary figures [file phytokeys-248-041_article-132707__-s002.zip › Suppl2/132707_1C-1-A_Figure_S1.jpg]

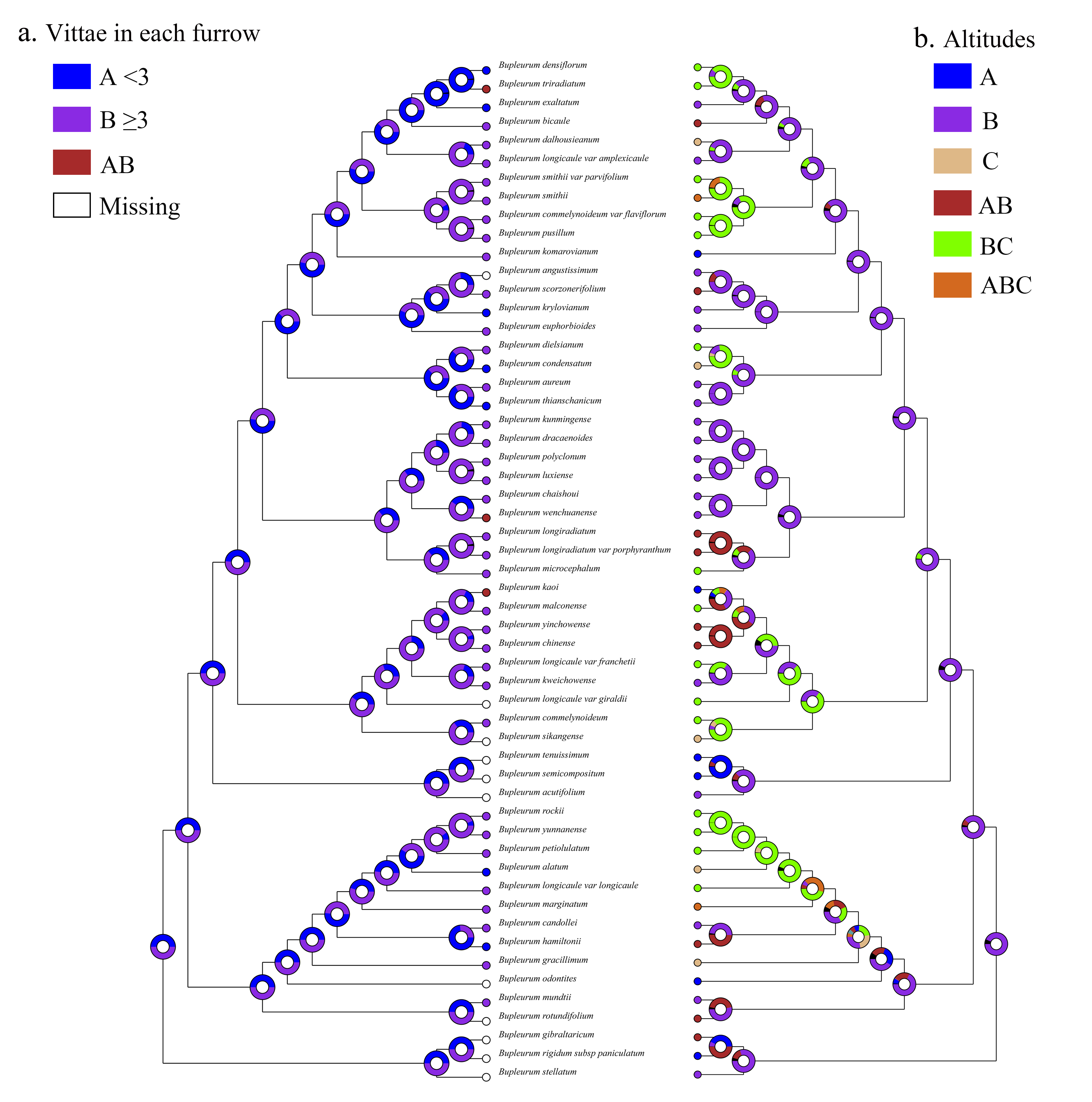

Supplement: Supplementary material 2 — Supplementary figures [file phytokeys-248-041_article-132707__-s002.zip › Suppl2/132707_1C-1-A_Figure_S5.jpg]

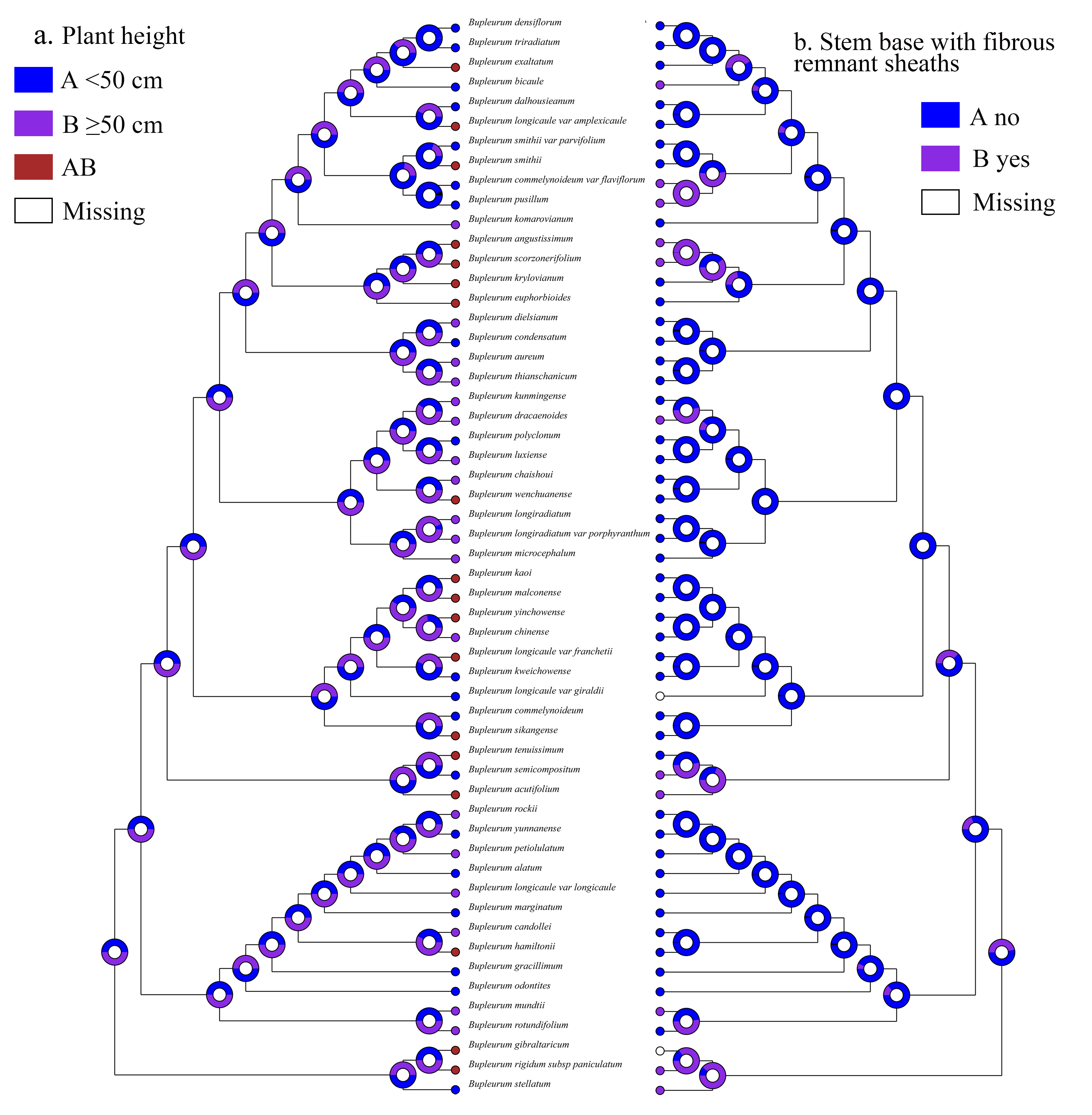

Supplement: Supplementary material 2 — Supplementary figures [file phytokeys-248-041_article-132707__-s002.zip › Suppl2/Figure S2.jpg]

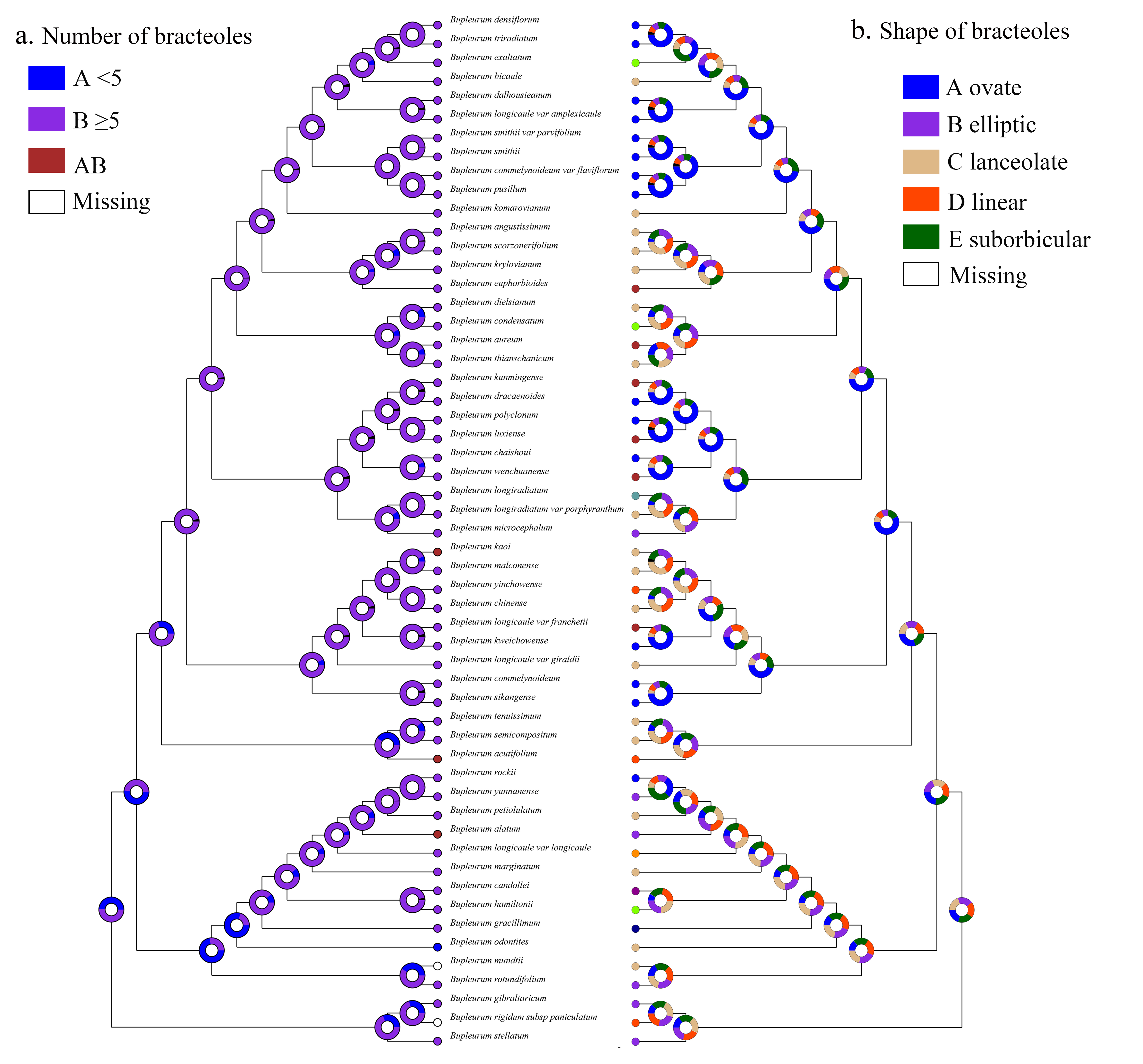

Supplement: Supplementary material 2 — Supplementary figures [file phytokeys-248-041_article-132707__-s002.zip › Suppl2/Figure S3.jpg]

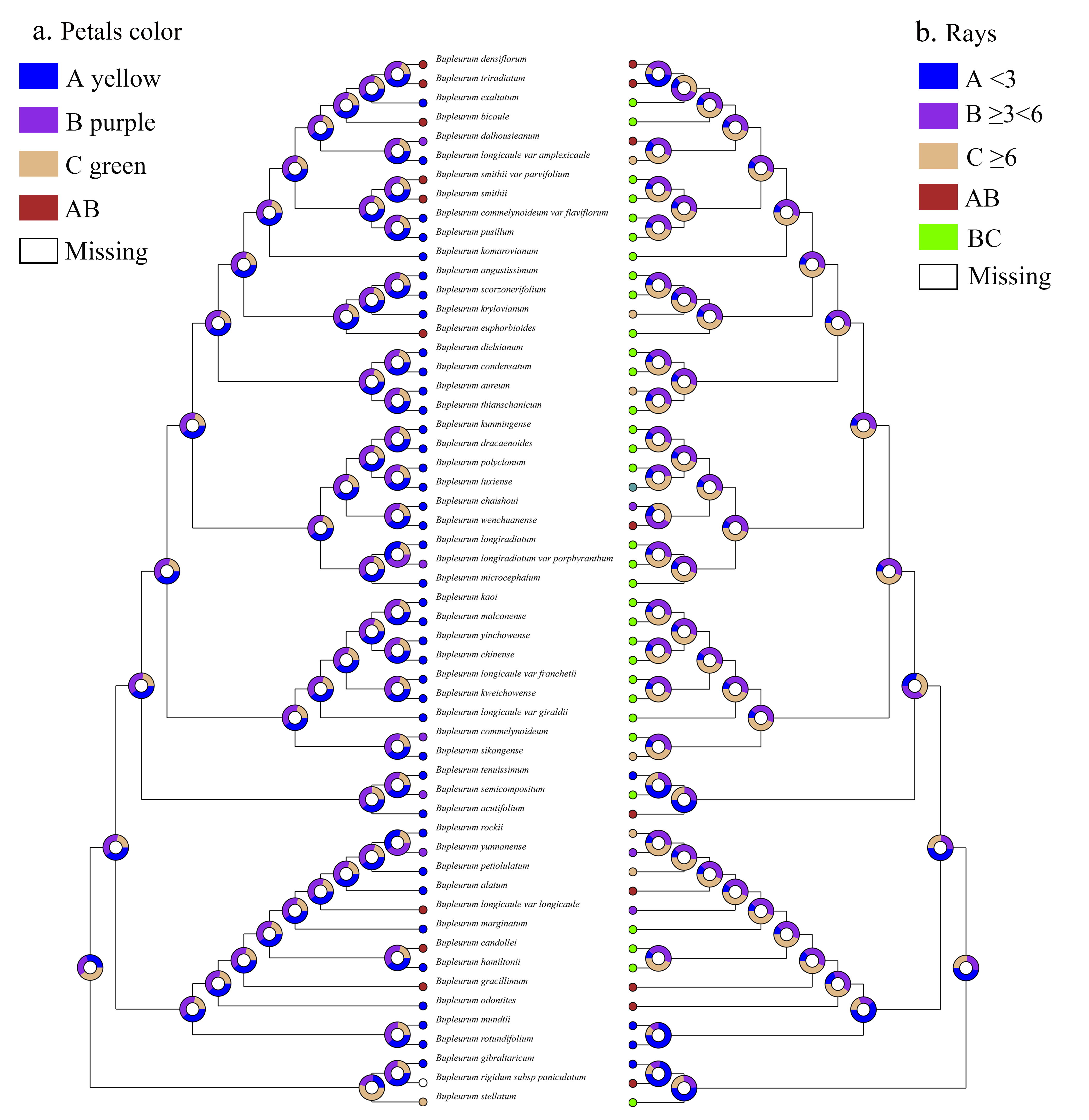

Supplement: Supplementary material 2 — Supplementary figures [file phytokeys-248-041_article-132707__-s002.zip › Suppl2/Figure S4.jpg]
